# Assessing Social – Ecological Trade-Offs to Advance Ecosystem-Based Fisheries Management

**DOI:** 10.1371/journal.pone.0107811

**Published:** 2014-09-30

**Authors:** Rudi Voss, Martin F. Quaas, Jörn O. Schmidt, Olli Tahvonen, Martin Lindegren, Christian Möllmann

**Affiliations:** 1 Department of Economics, University of Kiel, Kiel, Germany; 2 Kiel Institute for the World Economy, Kiel, Germany; 3 Department of Forest Sciences, University of Helsinki, Helsinki, Finland; 4 Scripps Institution of Oceanography, University of California San Diego, San Diego, California, United States of America; 5 Institute for Hydrobiology and Fisheries Science, Center for Earth System Research and Sustainability (CEN), University of Hamburg, Hamburg, Germany; Institut Maurice-Lamontagne, Canada

## Abstract

Modern resource management faces trade-offs in the provision of various ecosystem goods and services to humanity. For fisheries management to develop into an ecosystem-based approach, the goal is not only to maximize economic profits, but to consider equally important conservation and social equity goals. We introduce such a triple-bottom line approach to the management of multi-species fisheries using the Baltic Sea as a case study. We apply a coupled ecological-economic optimization model to address the actual fisheries management challenge of trading-off the recovery of collapsed cod stocks versus the health of ecologically important forage fish populations. Management strategies based on profit maximization would rebuild the cod stock to high levels but may cause the risk of stock collapse for forage species with low market value, such as Baltic sprat (Fig. 1A). Economically efficient conservation efforts to protect sprat would be borne almost exclusively by the forage fishery as sprat fishing effort and profits would strongly be reduced. Unless compensation is paid, this would challenge equity between fishing sectors (Fig. 1B). Optimizing equity while respecting sprat biomass precautionary levels would reduce potential profits of the overall Baltic fishery, but may offer an acceptable balance between overall profits, species conservation and social equity (Fig. 1C). Our case study shows a practical example of how an ecosystem-based fisheries management will be able to offer society options to solve common conflicts between different resource uses. Adding equity considerations to the traditional trade-off between economy and ecology will greatly enhance credibility and hence compliance to management decisions, a further footstep towards healthy fish stocks and sustainable fisheries in the world ocean.

## Introduction

A central issue in ecosystem-based management (EBM) is to identify potential trade-offs among multiple ecosystem goods and services [1]. The science underlying EBM has gained a lot of interest in the scientific literature [2], and concepts regarding evaluation of trade-offs [3], and for cross-sectorial approaches exist [4], [5]. However, there is no consensus among the expert community concerning the question, which factors need to be considered in EBM and to which depth. This has caused a lack of scientific agreement on how to implement EBM and, consequently, implementation is largely lacking. This is exemplified by fisheries management that in many parts of the world, and the European Union (EU) in particular, is still conducted on a species-by-species basis, as studies showing the importance of direct and indirect species interactions in marine food webs might have not been adequately build into the advice process and have not been accommodated by managers [6]–[8]. Furthermore, any integration of existing social-ecological knowledge and ecological-economic modeling is missing and accordingly can't be used during the decision making process, despite fisheries being a profoundly social and economic enterprise.

A challenge of EBM lies in balancing a number of potentially conflicting interests related to resource use, their equitable distribution and conservation. Such “triple-bottom line” solutions are commonly seen as the ideal outcome of conservation and management [9]. However, while conservation planning is now beginning to consider equity [10], issues of socio-economic equity have not been adequately addressed in fishery management plans [11], [12]. This is unfortunate, because management that fails to consider the fair distribution of benefits that ecosystems provide, e.g. equity in allocation of fishing rights, causes low acceptance and compliance [12]–[14] and ultimately overfishing through illegal, unregulated and unreported (IUU) fishing [15], [16].

Here, we provide a practical example on how to advance fisheries management towards an EBM approach by analyzing social-ecological trade-offs in a multi-species fisheries system. As an illustrative case study, we address the trade-off between recovery of Atlantic cod (*Gadus morhua*) versus the health of ecologically important forage fish stocks in the Baltic Sea. Many of the cod stocks in the North Atlantic have suffered from overfishing and population collapse [17]–[20] with immense social and economic consequences [21]. Moreover, decimated cod stocks have caused increases in forage species populations [6]–[8]. Depending on the system, increasing forage populations are either relatively low-valued small to intermediate-sized pelagic fish species or high-valued shellfish populations like lobster or shrimp, e.g. in eastern Canada [22]. The economic value of forage species in relation to the value of predators will alter the trade-offs involved in decision-making. In the Baltic, population increase following the cod collapse was mainly observed in the low-valued stock of sprat. Besides being of direct commercial interest, forage species have an enormous indirect value as a primary food source for many marine top-predators targeted by fisheries [23], [24], as well as species of particular conservation and public concern, e.g., marine mammals and birds [25].

Cod recovery in the Baltic Sea [26], raises two fundamental fisheries management questions involving trade-offs: (i) How much biomass and potential economic yield, provided by the high value cod stocks, needs to be sacrificed to allow for the protection of lower market value, but ecologically important, forage fish species, and (ii) What are the additional costs of considering an equitable distribution of benefits between the demersal (cod) and pelagic (forage fish) fisheries sectors, given that the latter has expanded after the cod collapse?

Using a coupled ecological-economic optimization model framework we first derive the profit maximizing management solution for the entire multi-species fishery, including cod and the major Baltic Sea forage species herring (*Clupea harengus*) and sprat (*Sprattus sprattus*). Then, we explore two different management approaches for protecting the sprat stock for its ecological value, one based on profit maximization only, and an alternative considering equity between demersal and pelagic fishing sectors. Our work suggests that recovery strategies for cod (and potentially other depleted top-predators) may be very different when based on profit maximization alone, or when taking into account additional ecological and societal objectives, such as interacting species and fisheries rights (i.e. equity) during the planning process.

## Materials and Methods

### Ecological-economic model

We developed and applied a combined three-species, age-structured ecological-economic model, including the predatory *G. morhua* (cod) and the two forage fish species *C. harengus* (herring) and *S. sprattus* (sprat). Our model is an extension of a single-species age-structured fishery model [27]. Full detail of the model equations are given in the Supporting Information (Materials S1). The age-structured multi-species population dynamics are described as in standard fisheries stock assessment. For cod and herring we assume stock-recruitment functions of the Ricker type, for sprat we assume a Beverton-Holt type, thereby following the approach of [28]. Structuring a stronger density-dependence into the predator than in the prey dynamics reflects current ecological knowledge and implies a conservative estimate of optimal cod biomass in the simulations. Age-specific survival rates are constant for cod.

Residual (M1) and predation (M2) mortality estimates for the different age-classes of herring and sprat are based on regression analysis, using the output of a stochastic multi-species model SMS [29] on mortality for different stock sizes of cod. Predation mortality is almost linearly dependent on the cod stock biomass for a wide range of stock states [27]. This shortcut in calculation of M2 values was used to reduce model complexity and implies a dependency of predation mortality on both, predator and prey abundance. Data and estimation of model parameters are mainly based on International Council for the Exploration of the Sea (ICES) stock assessment data (ecological data) and the Scientific, Technical and Economic Committee for Fisheries (STECF) of the European Commission (economic data); they are given in detail in Table S1.

For modeling profits of the cod fishery, we use the specification from [30] with age-specific prices and a cost function of the Spence type [31]. Sprat and herring are modeled as schooling fisheries [27], where the market price is assumed to be independent of age.

For the multi-species setting, the objective is to maximize
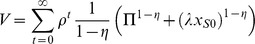
where 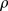 is the discount factor and 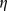 is the representative fisherman's aversion against intertemporal income fluctuations. The higher 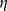 is, the more a constant income stream over time is preferred. Such a desire for relative constancy is reflected in several management plans for European fish stocks (e.g. Baltic cod [32]), which have been agreed upon by a broad range of stakeholders, including fishermen. It is expressed for example, as a requirement that total allowable catches (TACs) shall not change by more than a certain percentage between two subsequent years (15% in the case of Baltic cod).

The first part of this objective is the intertemporal utility of fishing income; where fishing income is a generalized mean of fishing incomes from the cod, sprat, and herring fisheries,
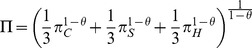



The parameter 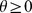
captures the social aversion against inequality of incomes for the three different fisheries. The higher 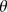 is, the more a constant income distribution is preferred. The second part of the objective captures the non-market benefits derived from ecosystem services provided by the sprat spawning stock 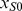, with 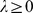 being the price (in Euros per kg of sprat spawning stock) society is willing to pay for these ecosystem services. With increasing λ the value of sprat ‘in the sea’ is rising; if λ reaches the shadow value of sprat, the fishery would be stopped. We determine the optimal management numerically, applying a dynamic optimization using the interior-point algorithm of the Knitro (version 8.0) optimization software with AMPL. Error bars for optimization results are obtained from a Monte-Carlo sensitivity analysis (Supporting Information, Materials S2 with Figures S1, S2) based on one standard error of estimated parameter values.

### Equity

We use the widely recognized Gini coefficient [33] that is often used in empirical work to describe equity in the distribution of profits between fisheries, i.e. between the cod, herring and sprat fisheries. As the Gini coefficient *per se* is a measure of inequality, we use 1 – the Gini coefficient to obtain a measure of equity. Ranging from 0 to 1, a value of 1 represents perfect equality, a value of zeo maximum inequality. Details on the calculation are given in the Supporting Information (Materials S1).

### Summary of multispecies management options

We summarize scenario-specific information on economic profits, conservation and social equity goals in a single graph. Modified pie charts include central numbers, which indicate total profits (million €/year) as well as an equity measure. The equity measure is based on the Gini index and is calculated as (1-Gini coefficient)*100. Pie slices give information on species-specific outcome. The size of each pie slice is calculated relative to status quo values 2008–2010 [34] on a cube-rooted axis.

## Results

### Profit maximization and the risk of forage fish stock collapse

**Figure 1:**
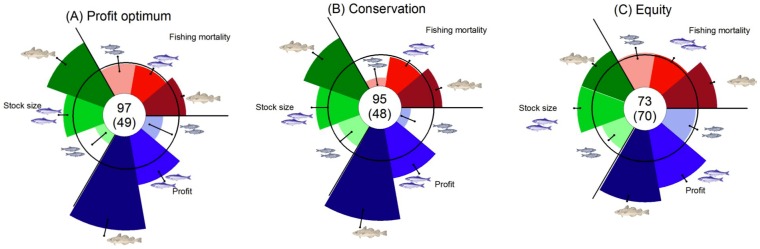
Summary of multispecies management options in the Baltic. (A) Profit maximum. (B) Economic optimum while respecting sprat B_PA_. (C) Equitable optimum while respecting sprat B_PA_. Central numbers indicate total profits (million €/year) as well as an equity measure (in brackets). Area of each pie slice is relative to status quo values 2008-2010 (black circle), with error bars from sensitivity analysis.

**Figure 2:**
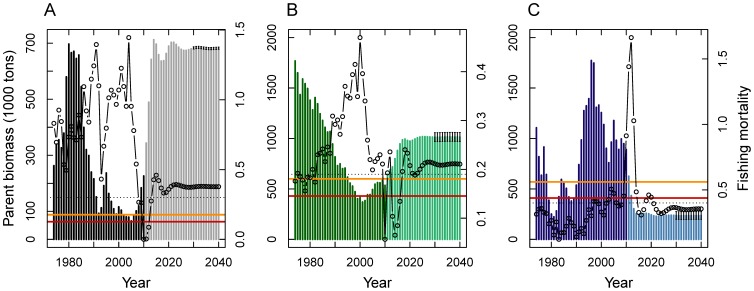
Profit maximizing management for the Baltic Sea multi-species fishery. Barplots show the time-trajectories of parent biomasses for cod (A), herring (B) and sprat (C). Darker bars represent the model initialization period (1974–2010), lighter bars the economically optimal solution from 2011 onwards. Error bars show the 95% confidence limits for steady state parent stock sizes from a Monte-Carlo sensitivity analysis with respect to predation mortalities; red and orange horizontal lines indicate ecological reference points B_lim_ and B_pa_ [34], respectively. Dots and line plots show the estimated fishing mortality coefficients. Dotted horizontal lines indicate current target fishing mortality coefficients. Values for reference points and target fishing mortality coefficients are given in Table S2.

We first applied our model framework to estimate the economically optimal steady-state strategy by maximizing the net present value of aggregate profits of the multi-species fishery. Simulations showed that a profit-maximizing multi-species management strategy may indeed lead to a full recovery of the once depleted cod stock, with parent biomass reaching levels close to the historical maximum of ∼700 thousand tons (Fig. 2A). The profit-maximizing solution revealed that a period of low fishing mortality (F), as presently observed, is necessary for the full recovery of the stock. The long-term F would be ∼0.4, hence even slightly higher than under the current EU (single-species) management plan (F = 0.3; [32]). Cod stock size in this scenario is well above recently determined multi-species management references levels [34] that indicate risk of overfishing, i.e. a precautionary limit (B_pa_) and the minimum biological acceptable limit (B_lim_) for parent stock biomass (Fig. 1A). It has to be noted, however, that status and reference points of the eastern Baltic cod stock are currently under debate.

Profit-maximizing multi-species harvesting would also result in a healthy and sustainable population size of herring (Fig. 2B), as the stock would recover to well above B_pa_ and B_lim_. After a recovery period with low fishing pressure, the equilibrium F would be slightly above 0.19, the F level that should lead to precautionary biomass levels (F_pa_) [35].

In contrast to cod and herring, profit maximizing multi-species management would increase the risk of sprat stock collapse, as the equilibrium stock size would fall largely below B_pa_ and B_lim_ (Fig. 2C), despite low equilibrium F. This outcome would be due to the higher market value of cod (compared to the forage species; Table S1), that favors cod recovery and hence higher predation pressure, lower sprat biomass and poor economic return to the forage fishing sector.

### Valuing conservation goals

**Figure 3:**
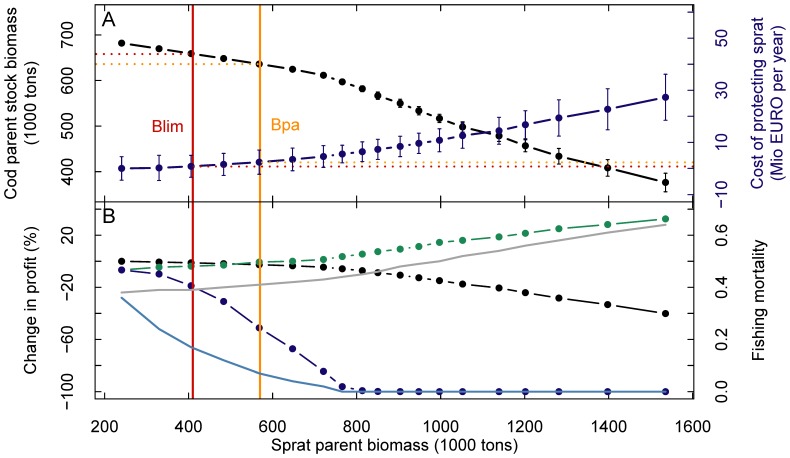
Conservation of sprat through its ecosystem value. (A) Trade-off between sprat and cod parent biomass (black dots and lines) and costs for the overall Baltic fishery of maintaining set levels of sprat parent stock size (blue line). Error bars show standard errors from a Monte-Carlo sensitivity analysis with respect to predation mortalities. (B) Dot and line plots show the percentage change in fishery-specific steady-state profits as a result of maintaining set levels of sprat parent stock size (cod – black, herring – green, sprat – blue). Lines show fishing mortality coefficients (cod – grey, sprat – blue) required to achieve respective sprat stock sizes in steady state. Red and orange vertical lines show ecological reference points B_lim_ and B_pa_ for sprat [34].

Sprat has a key role in the Baltic Sea food-web as prey for cod [36], marine mammals [37], and birds [38]. Hence, depleting the sprat stock bears unforeseeable risks for ecosystem functioning, service provision and protection of species with particular conservation concern. In economic terms these are externalities that should be taken into account when designing socially reasonable policies. We evaluated the consequences of protecting the sprat stock for its ecosystem value by performing multiple model simulations (Fig. 2) during which we varied the social willingness to pay for parent biomass of sprat (the shadow price of the externality). The resulting relationships between sprat parent biomass and variables of the other two species represent *efficiency frontiers*, providing management options for the optimal delivery of conflicting services [10]; [39], [40]. Following the typology of [3] the interaction between sprat and cod parent stock sizes is concave. To achieve sprat stock sizes corresponding to B_lim_ and B_pa_, only a minor reduction of cod parent biomass would be necessary, i.e., by 4 or 7% relative to the profit optimum of 682 thousand tons, respectively (Fig. 3A). Overall this management strategy would cause a potential loss of profit for the combined Baltic Sea fishery amounting to 0.8 or 2.4 M€, corresponding to 0.8 and 2.5% relative to the economically optimal management solution in the steady state.

While this management strategy would only marginally affect cod and herring profits (Fig. 3B), the relationship between sprat biomass and sprat profit is strongly convex [3], meaning that over the small range between B_lim_ and B_pa_, and above, the profit of the sprat fishery would collapse. Increasing sprat biomass in the steady state to B_lim_ and B_pa_ would need a reduction of the sprat fishing mortality to 0.17 and 0.07 (from 0.36 in the profit optimum), respectively, causing potential sprat profit losses of 13 of 48%. At the same time, cod fishing mortalities are less affected and would need to be increased only to 0.39 and 0.41 for B_lim_ and B_pa_, respectively (from 0.38 in the profit optimum). The cod fishing sector would only loose 1.2 or 2.6% of its potential profit. This result indicates that under an economic optimization, as performed here, the economically efficient solution to protect the sprat stock is a pronounced direct reduction of the fishing pressure on this lower market value forage fish species, in combination with a minor increase in fishing pressure directed towards its predator.

Clearly, the conservation strategy of increasing the sprat stock by directly decreasing the sprat fishing mortality would be ecologically and economically efficient, since it requires only a minor reduction of the cod stock and hence has only a minor effect on the highly profitable cod fishing sector. But, while the pelagic herring fishery sector would benefit from a slight increase in profits (Fig. 3B), the sprat fishery would be marginalized, with sprat fishing license holders carrying almost the complete costs of the conservation effort. It is doubtful that such a management strategy would find acceptance by the presently expanded pelagic fishing sector, unless compensation payments are made between the different fisheries. A practical implementation of compensation schemes between fisheries is likely to be difficult or even infeasible. However, it might depend greatly on the incentives and alternatives available.

### Conservation considering equitable resource distribution

**Figure 4:**
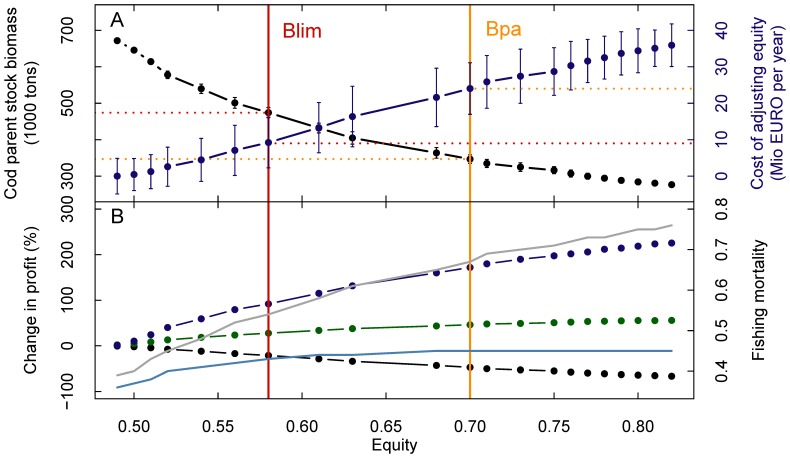
Conservation of sprat through equitable profit distribution for the three fisheries. (A) Trade-off between equity and cod parent biomass (black dots and lines) and costs for the overall Baltic fishery of maintaining set levels of equity between profits for the three fisheries (blue line). Error bars show 95% standard errors from a Monte-Carlo sensitivity analysis with respect to predation mortalities. (B) Dot and line plots show the percentage change in fishery-specific steady-state profits as a result of deriving set levels of equity (cod – black, herring – green, sprat – blue). Lines show fishing mortality coefficients (cod – grey, sprat – light blue) required to achieve respective equity levels. Red and orange vertical lines show equity levels required to achieve sprat parent stock sizes at the ecological reference points B_lim_ and B_pa_ for sprat [34].

An alternative to apply an increasing value to the conservation of the sprat stock is to explore the consequences of an increasing equitable resource distribution between fishing sectors. We defined equity based on relative profits of the three interacting species using the Gini-Index (see methods and Materials S1), and optimized the multi-species model for increasing equity levels (Fig. 4). Increasing equity corresponds to increasing fishing opportunities for sprat license holders and hence requires an increasingly larger sprat, but a reduced cod stock. We found a slightly convex efficiency frontier [3] for this trade-off, i.e. increasing equity to achieve sprat stock sizes in a range roughly corresponding to B_lim_ and B_pa_ would require a strong reduction of optimal cod stock sizes to c. 474 and 346 thousand tons, respectively (Fig. 4A). However, these estimates are still above the present stock size of c. 211 thousand tons [35], as well as B_lim_ and B_pa_ [34]. Overall increasing equity is positively linearly related to costs for the combined Baltic Sea fishery, which would amount to a loss of c. 9 or 24 Mio € per year (for B_lim_ and B_pa_, sprat parent biomasses respectively) relative to the profit-maximizing multi-species solution.

Naturally reduced profits of the high value cod fishery make up for most the conservation costs inherent in the management strategy considering equity (Fig. 4B). Cod profit losses would amount to c. 21 or 47% of the potential profit at sprat B_lim_ and B_pa_, respectively. The sprat fishing sector would achieve c. 92% or 172% higher profits at sprat B_lim_ and B_pa_, respectively, compared to the profit-maximizing multi-species solution, while the effect on herring profits would be negligible.

Increased equity between fishing sectors can only be achieved by a lowered predation pressure on sprat and hence a reduction of the cod stock due to a stronger fishing pressure (Fig. 4B). Achieving levels of equity that lead to sprat biomasses at B_lim_ or B_pa_ levels would need an increase in cod F to 0.54 and 0.67 (from 0.38 in the profit maximizing scenario). Historical evidence suggests that the cod stock biomass would still be sustainably conserved under such fishing pressure.

## Discussion

Our case study from the Baltic Sea revealed that EBM approaches to fisheries require model systems that account for multi-species trophic interactions and have the ability to link ecology and economy [20], [41]. Our multi-species model consequently challenges traditional single-species approaches since optimal, long-term stock sizes and profits are significantly smaller compared to species-by-species simulations (see Supporting Information, Materials S3 with Figure S3). However, our model framework has room for improvements, in particular regarding environmental influences on recruitment [42], density-dependent growth [43], [44] and processes accounting for changes in the spatio-temporal overlap of cod and sprat [45]. Future work should analyze which further information is most needed to support decision-making, and which factors have been associated with weaker management decisions in the past. Nevertheless, we are confident in the range of simulated outcomes, e.g., confirming a strong recovery potential for Baltic cod [20], [46]–[48]. The conclusions might, however, change, if the ecological system as a whole undergoes substantial changes and historic relationships concerning predation rates and/or stock dynamics will no longer hold. Presently, expert opinions on the state of the eastern Baltic cod stock and its recovery potential diverge [49]. A possible (density-dependent) decrease in cod ability to capture prey, and the resulting hysteresis – an even lower foraging ability due to the lack of food – might alter the qualitative conclusions.

Our study area has been shown to be well suited for addressing applied ecological questions relevant to EBM [7], [20], [50], [51], and we show that it is a suitable case study for demonstrating the principles of trade-off evaluation in multi-species fisheries. The Baltic has a comparatively simply food-web with strong, quantifiable predator-prey relationships, and fishing fleets target mainly single species. Therefore, key ecological, economic, and equity trade-off characteristics can relatively easy be expressed and captured in an integrated model. This might be more difficult when trying to extrapolate these methods to other more complex (in ecological as well as economic terms) systems. We are, however, confident that our approach is readily transferrable to other systems, since quantitative predator-prey models as well as dynamic fleet models capturing the key characteristics of more complex systems are becoming increasingly available [52], [53], forming the basis for reliable coupled ecological-economic models.

Our results confirm that triple-bottom-line management solutions are usually costly [10]. Protecting the sprat stock for its ecosystem value in an economic efficient way that disregards equity between fishing sectors would only have minor consequences for the cod stock and low costs for the overall multi-species fishery (Table 1). The economically preferable approach would be to implement this management strategy together with a scheme of transfer payments that compensate the sprat fishery for forgone potential profits. In practice, such transfer payments may lead to sustained over-capacity in the fishery and have been criticized. If such a compensation scheme is not feasible, solving the emergent social conflict by achieving equity between fishing sectors would require to sacrifice a larger part of the cod stock as well as harvest, and hence economic potential of the Baltic Sea fishery as a whole. However, a triple-bottom-line solution, that for example has the goal to maintain the Baltic Sea sprat stock at the recently determined precautionary biomass reference level B_pa_ while at the same time maintaining the equity level, may provide a reasonable compromise, i.e., a zone of ‘new consensus’ [54] for the whole multi-species system. This management option minimizes the risk of forage fish overfishing and assures the viability of the pelagic fishing sector. Although the cod fishing sector would lose a considerable amount of potential profit, our most equitable solution still allows for ongoing growth of the cod fishery, offering a potential win-win situation over all fishing sectors. When invoking the notion of “equity” one has to bear in mind that the value chains for capture and processing are different between the pelagic forage fishery and the cod fishery. Value chains for the forage fishery are usually highly centralized, with a need for significant capital investment, infrastructure, and large scale marketing, while in the cod fishery the harvester can take over large parts of these activities. Unfortunately, we currently do not have enough data to apply equity analysis for the whole value chain in the Baltic fisheries, but we acknowledge that this would turn the equity issue even more complex. It has to be noted that the steady-state cod fishing mortalities for the equity maximizing management option are below mean historical levels (Fig. 1A), but higher compared to the presently enforced long-term management plan (Table 1). While this reference level is deliberately conservative, our results may be due to a high steady-state cod stock biomass which may prove overestimated, at least during unfavorable climate conditions for cod recruitment [55]. Hence, a critical evaluation using model ensemble approaches is warranted [44]. However, the level of fishing would be well below the long-term average F before adoption of the long-term cod management plan in 2006.

**Table 1:** Effects of management strategies for the conservation of sprat.

Strategy	SPB		Equity	
Reference Point	B_pa_	B_lim_	B_pa_	B_lim_
Decrease in cod parent biomass (%)	−7	−4	−47	−21
Costs to the Baltic fishery (Million $)	2.5	0.8	24	9
Sprat fishing mortality	0.07	0.17	0.41	0.39
Cod fishing mortality	0.45	0.43	0.67	0.54

Effect on cod biomass, costs for the overall Baltic fishery, cod and sprat steady state fishing mortality coefficients. Equity – management strategy considering equitable resource distribution, SPB – management strategy through profit maximization at set **S**prat **P**arent **B**iomass.

Last but not least, operationally applying ecological-economic models systems in a way demonstrated in our study will facilitate coordinated management decisions among interacting use sectors as well as stakeholder involvement, both critical components in EBM approaches leading to increased societal values of exploited ecosystems [56]. Through this approach another aspect of equity, i.e. participatory equity, is addressed which increases the acceptability and hence compliance to management decisions [10], a further footstep towards healthy fish stocks and sustainable fisheries in the world ocean.

## Supporting Information

Materials S1 **Ecological-economic model, equity, and programming code.**
(DOC)

Materials S2 **Sensitivity analysis.** Including Figure S1 (Relationship between the parameter of the welfare function [measured in Euros per kg of sprat spawning stock biomass] and the resulting steady-state spawning stock biomass of sprat), as well as Figure S2 (Relationship between the parameter of the welfare function and the resulting steady-state spawning stock biomass of sprat (a), and between the parameter of the welfare function and the resulting steady-state equity, as measured by 1 – the GINI coefficient.
(DOCX)

Materials S3 **Single versus multi-species management**. Including Figure S3 (Timepath of optimal singlespecies management (red lines) in terms of spawning stock size (full line) and profit (dotted line) versus optimal multispecies management (blue lines); data from profit maximizing model run 2010-2040; trade-offs between cod and sprat (left panel), cod and herring (middle) and herring and sprat (right).
(DOCX)

Table S1 **Parameters of the multi-species ecological-economic model.** Subscripts C, S, H refer to cod, sprat and herring, respectively.
(DOCX)

Table S2 **Biomass limits using the precautionairy approach (Bpa) and limit biomass levels (Blim) as well as different target fishing mortality rates depending on availability.**
(DOCX)
