# Supplementary material for: Assessing Social – Ecological Trade-Offs to Advance Ecosystem-Based Fisheries Management
Source: PLoS One. 2014 Sep 30;9(9):e107811. doi: 10.1371/journal.pone.0107811 (PMC4182428; doi:10.1371/journal.pone.0107811)
Supplement: Materials S1 — Ecological-economic model, equity, and programming code. (DOC) [file pone.0107811.s001.doc]

**Materials S1: Ecological-economic model, equity, and programming cod**

*Ecological-economic model*

We developed and applied a combined three-species, age-structured ecological-economic model the predatory cod (*Gadus morhua*) and the two forage fish species herring (*Clupea harengus*) and sprat (*Sprattus sprattus*). Our model is an extension of a single-species age-structured fishery model (Tahvonen 2009; Tahvonen et al. 2013).

We use $x_{ist}$ to denote the number of fish of species $i\in\{C,S,H\}$, where *C*  stands for cod, *S* for sprat, and *H* for herring, in age group $s=1,\ldots, n$ and at the beginning of period $t=0, 1,\ldots$. We use $\alpha_{is}>0$, $i\in\{C,S,H\}$, $s=1,\ldots, n$ to denote age specific survival rates, $\gamma_{is}>0$, $i\in\{C,S,H\}$, $s=1,\ldots, n$ to denote age specific proportions of mature individuals and $w_{is}$, $i\in\{C,S,H\}$, $s=1,\ldots, n$ to denote the mean weights (in kilograms) of fish of species $i\in\{C,S,H\}$ in age group $s=1,\ldots, n$. For cod, all of these parameters are assumed to be constant (Tahvonen 2009), and as in the standard biological stock assessments for the Eastern Baltic cod (ICES 2012). For sprat and herring, we assume that proportions of mature individuals and weights are constant, but the survival rates of sprat depend on cod spawning stock biomass. We use the specification

$$\alpha_{is}=\exp\left(-M_{2is}-\delta_{is} x_{C0t}\right)$$

where *M_2is_* is instantaneous natural mortality of sprat cohort *s* in the absence of cod, and $\delta_{is}$is a positive parameter that measures the dependency of instantaneous natural mortality of sprat (i=S) and herring (i=H) cohort *s* on cod spawning stock biomass, $x_{C0t}$.

Denoting the recruitment function for species $i\in\{C,S,H\}$ by φ_i_ ( ) and the spawning biomass by $x_{i0t}$, the age structured population model with harvesting activity can be summarized as

Where $h_{ist}$is the number of fish harvested from cohort *s* of species *i* in period *t*.

We assume perfect selectivity with respect to the species, which is a reasonable assumption for the Baltic, as different species are caught by different fleets.

Aggregate instantaneous fishing mortality $F_{it}$for species *i* in year *t* translates into age-specific fishing mortalities, captured by the constant, age-specific catchability coefficients $q_{is}\geq0$, $i\in\{C,S,H\}$, $s=1,\ldots, n$, such that

$h_{ist}=q_{is}x_{ist}\left(1-\exp\left(-F_{it}\right)\right)$.

For cod and herring we assume stock-recruitment functions of the Ricker (1954) type (Quaas et al. 2013), i.e. we assume for $i\in\{C,H\}$. For sprat we assume a Beverton-Holt type (Tahvonen et al. 2013), i.e. we assume .

For modeling profits of the cod fishery, we use the specification from (Quaas et al. 2012) with age-specific prices and a cost function of the Spence-type (Spence 1974). Thus, profits $\pi_{Ct}$ of the cod fishery in year *t* are

Where *p_Cs_* are prices, *w_Cs_* are weights, and *q_Cs_* are catchabilities for cod in age group *s*; *F_Ct_* is instantaneous fishing mortality (i.e., instantaneous effort), and *c_C_* is the unit effort cost for the cod fishery.

Sprat and herring are modeled as schooling fisheries (Tahvonen et al. 2013), where the market price *p_i_* is assumed to be independent of age. The profits in the sprat and herring fisheries thus are

$$\pi_{it}=\sum_{s=1}^{n} \left(p_{i}-c_{i}\right)h_{ist}$$

With analogous interpretations for the symbols as for the cod fishery.

For the single-species optimization, the objective is to maximize a representative fisherman’s intertemporal utility from fishing income,

$$V_{i}=\sum_{t=0}^{\infty} \rho^{t}\frac{\pi_{i}^{1-\eta}}{1-\eta}$$

where $\rho$ is the discount factor and $\eta$ is the representative fisherman’s aversion against intertemporal income fluctuations. The higher $\eta$ is, the more a constant income stream over time is preferred. Such a desire for relative constancy is reflected in several management plans for European fish stocks (e.g. Baltic cod; EC 2007), which have been agreed upon by a broad range of stakeholders, including fishermen. It is expressed for example, as a requirement that total allowable catches (TACs) shall not change by more than a certain percentage between two subsequent years (15% in the case of Baltic cod).

For the multi-species setting, the objective is to maximize

$$V=\sum_{t=0}^{\infty} \rho^{t} \frac{1}{1-\eta} \left(\Pi^{1-\eta}+\left(\lambda x_{S0}\right)^{1-\eta}\right)$$

The first part of this objective is similar to the single-species objective: It is the intertemporal utility of fishing income; where fishing income is a generalized mean of fishing incomes from the cod, sprat, and herring fisheries,

$$\Pi=\left(\frac{1}{3}\pi_{C}^{1-\theta}+\frac{1}{3}\pi_{S}^{1-\theta}+\frac{1}{3}\pi_{H}^{1-\theta}\right)^{\frac{1}{1-\theta}}$$

The parameter $\theta\geq0$captures the social aversion against inequality of incomes for the three different fisheries. The higher $\theta$ is, the more a constant income stream over time is preferred.

To measure **equity,** we use the widely recognized Gini coefficient that is often used in empirical work to describe equity in the distribution of profits between fisheries, i.e. between the cod, herring and sprat fisheries. The Gini coefficient has pleasing properties, for example it is Lorenz-consistent. As the Gini coefficient per se is a measure of inequality, we use 1 – the Gini coefficient to obtain a measure of equity (Halpern et al. 2013). To illustrate how it is calculated, assume that the sprat fishery has the smallest profit, and the cod fishery the largest. Then, the equity measure is

$$E=\frac{5\pi_{S}+3\pi_{H}+\pi_{C}}{3\left(\pi_{S}+\pi_{H}+\pi_{C}\right)}$$

Ranging from 0 to 1, a value of 1 represents perfect equality, a value of zero maximum inequality.

*Data and parameterization of the model*

Data and estimation of model parameters are mainly based on International Council for the Exploration of the Sea (ICES) stock assessment data (ecologic data) and the Scientific, Technical and Economic Committee for Fisheries (STECF) of the European Commission (economic data).

For all species, we consider 8 age groups, as in the ICES standard assessments for the Baltic fish stocks. Age-specific parameter values are reported in Table S1. Maturities, and natural mortalities for cod, are directly taken from the ICES stock assessment reports. For age-specific weights, we use the weight in stock for 2010, and for the initial stock numbers we use the figures from the stock assessment reports (ICES 2012). Catchabilities are based on mean age-specific fishing mortalities for the years 2006–2010 reported in (ICES 2012), with *q*_is_ = 1 for age group with the highest fishing mortality by normalization. Natural mortality estimates used in the ecological-economic modeling. Residual and predation mortality estimates for the different age-classes of herring and sprat are based on regression analysis, using SMS output on mortality for different stock sizes of cod and are reported in Table S1.

For the parameters of the stock-recruitment function we use the estimates of (Quaas et al. 2013) for cod, i.e. we use and For sprat, we use the estimates of (Tahvonen et al. 2013), which are and Finally, for herring we use and Herring parameters were obtained by fitting a model to data from 1974 to 2011 as provided by (ICES 2012).

The cost parameter for the cod fishery is *c_C_* = 55.2 million euros (Quaas et al. 2012). For cod, age-specific prices are reported in Table S1. For sprat, we use a net price (i.e. price net of marginal harvesting costs) *p_S_-c_S_=*0.039 (Tahvonen et al. 2013); for herring, we use *p_H_-c_H_*=0.100 (Quaas et al. 2012). We further specify $\eta=0.25$ .

The time horizon for the optimization is 100 years, and we use a discount rate of 1 percent p.a.

*Optimization and programming codes*

We determine the optimal management numerically, applying a dynamic optimization using the interior-point algorithm of the Knitro (version 8.0) optimization software with AMPL. Programming codes for the dynamic optimization are provided in the following.

File Baltic.run

reset;

model Baltic.mod;

data Baltic.dat;

# define intial point for solution

let {i in 1..3, s in 1..n, t in 1..T} x[i,s,t] := xstart[i,s];

let {i in 1..3, t in 0..T} F[i,t] := 0.3;

let lambda := 0;

let theta := 0.05;

option solver "knitroampl";

option knitro_options 'maxit=3000 opttol=1.0e-9';

solve;

printf "lambda %f\t", lambda>Results.csv;

printf "theta %f\n", theta>Results.csv;

printf "year\t">Results.csv;

printf "ssb [1000 tons]\t\t\t">Results.csv;

printf "catch [1000 tons]\t\t\t">Results.csv;

printf "fishing mortality\t\t\t">Results.csv;

printf "profit [million euros]\t\t\n">Results.csv;

printf "\t cod\t">Results.csv;

printf "sprat\t">Results.csv;

printf "herring\t">Results.csv;

printf "cod\t">Results.csv;

printf "sprat\t">Results.csv;

printf "herring\t">Results.csv;

printf "cod\t">Results.csv;

printf "sprat\t">Results.csv;

printf "herring\n">Results.csv;

for {i in 0..40}

{

printf "%f\t", i+2010>Results.csv;

printf "%f\t", x0[1,i]>Results.csv;

printf "%f\t", x0[2,i]>Results.csv;

printf "%f\t", x0[3,i]>Results.csv;

printf "%f\t", H[1,i]>Results.csv;

printf "%f\t", H[2,i]>Results.csv;

printf "%f\t", H[3,i]>Results.csv;

printf "%f\t", -log(1-F[1,i])>Results.csv;

printf "%f\t", -log(1-F[2,i])>Results.csv;

printf "%f\t", -log(1-F[3,i])>Results.csv;

printf "%f\t", profit[1,i]>Results.csv;

printf "%f\t", profit[2,i]>Results.csv;

printf "%f\n", profit[3,i]>Results.csv;

}

end

File Baltic.mod

param T; #time horizon (years)

param n; #number of age classes

param r; #annual interest rate

param w {i in 1..3, s in 1..n}; #weight; unit kg per individual in age class

param gamma {i in 1..3, s in 1..n}; #maturity

param q {i in 1..3, s in 1..n}; #selectivity

param M2 {i in 1..3, s in 1..n}; #natural mortality rates

param delta {i in 1..3, s in 1..n}; #predation mortality parameter

param xstart {i in 1..3, s in 1..n}; #initial state, number of individuals

param p {i in 1..3, s in 1..n}; #price

param c {i in 1..3}; #cost parameter for cod

param eta; #intertemporal inequality aversion

param theta; #inequality aversion between fisheries

param lambda; #preference for ecosystem services

param phi1 {i in 1..3}; #parameters of stock-recruitment functions

param phi2 {i in 1..3};

var F {i in 1..3, t in 0..T}>=0<=0.95; #Fishing mortality

var x {i in 1..3, s in 1..n,t in 0..T}>= 0; #number of individuals [millions]

var x0 {i in 1..3, t in 0..T-1}=sum{s in 1..n} w[i,s]*gamma[i,s]*x[i,s,t] #spawning stock [1000 tons]

var H {i in 1..3, t in 0..T}= sum{s in 1..n} q[i,s]*w[i,s]*x[i,s,t]*F[i,t];

var profit {i in 1..3, t in 0..T}= sum{s in 1..n} p[i,s]*q[i,s]*w[i,s]*x[i,s,t]*F[i,t]+c[i]*log(1-F[i,t]);

maximize objective_function: sum{t in 0..T} (1/(1+r))^t*(((1/3)*profit[1,t]^(1-theta)+(1/3)*profit[2,t]^(1-theta)+(1/3)*profit[3,t]^(1-theta))^((1-eta)/(1-theta))+(lambda*x0[2,t])^(1-eta));

#recruitment

subject to constraint1COD {t in 0..T-1}: x[1,1,t+1]=phi1[1]*x0[1,t]*exp(-x0[1,t]/phi2[1]);

subject to constraint1SPR {t in 0..T-1}: x[2,1,t+1]=phi1[2]*x0[2,t]/(phi2[2]+x0[2,t]);

subject to constraint1HER {t in 0..T-1}: x[3,1,t+1]=phi1[3]*x0[3,t]*exp(-x0[3,t]/phi2[3]);

#population dynamics

subject to constraint2COD {s in 1..n-2, t in 0..T-1}: x[1,s+1,t+1]=exp(-M2[1,s])*(1-q[1,s]*F[1,t])*x[1,s,t];

subject to constraint3COD {t in 0..T-1}: x[1,n,t+1]=exp(-M2[1,n-1])*(1-q[1,n-1]*F[1,t])*x[1,n-1,t]+exp(-M2[1,n])*(1-q[1,n]*F[1,t])*x[1,n,t];

subject to constraint2CLU {i in 2..3, s in 1..n-2, t in 0..T-1}: x[i,s+1,t+1]=exp(-M2[i,s]-delta[i-1,s]*x0[1,t])*(1-q[i,s]*F[i,t])*x[i,s,t];

subject to constraint3CLU {i in 2..3, t in 0..T-1}: x[i,n,t+1]=exp(-M2[i,n-1]-delta[i-1,n-1]*x0[1,t])*(1-q[i,n-1]*F[i,t])*x[i,n-1,t]+exp(-M2[i,n]-delta[i-1,n]*x0[1,t])*(1-q[i,n]*F[i,t])*x[i,n,t];

#initial stock numbers

subject to initial_condition {i in 1..3, s in 1..n}: x[i,s,0] = xstart[i,s];

File Baltic.dat

param T := 100;

param n := 8;

param r := 0.01;

param c :=

1 55.2

2 0.0

3 0.0;

param p :=

1 1 0.0

1 2 0.35

1 3 0.35

1 4 0.35

1 5 0.477

1 6 0.477

1 7 0.636

1 8 0.731

2 1 0.039

2 2 0.039

2 3 0.039

2 4 0.039

2 5 0.039

2 6 0.039

2 7 0.039

2 8 0.039

3 1 0.100

3 2 0.100

3 3 0.100

3 4 0.100

3 5 0.100

3 6 0.100

3 7 0.100

3 8 0.100;

param eta := 0.25;

param phi1 :=

1 1.70

2 104.2

3 30.3;

param phi2 :=

1 549

2 503.2

3 2156;

param w:=

1 1 0.08

1 2 0.187

1 3 0.698

1 4 0.85

1 5 1.022

1 6 1.258

1 7 2.218

1 8 3.792

2 1 5.2

2 2 8.0

2 3 9.9

2 4 10.7

2 5 11.0

2 6 11.2

2 7 10.8

2 8 11.4

3 1 0.012

3 2 0.0183

3 3 0.0258

3 4 0.0322

3 5 0.0332

3 6 0.0385

3 7 0.045

3 8 0.045;

param q :=

1 1 0

1 2 0.1234

1 3 0.5651

1 4 1.0000

1 5 0.9776

1 6 0.7797

1 7 0.6389

1 8 0.6389

2 1 0.4028

2 2 0.7695

2 3 0.8580

2 4 0.9643

2 5 0.8919

2 6 0.9965

2 7 1.0000

2 8 1.0000

3 1 0.1929

3 2 0.3896

3 3 0.6111

3 4 0.7881

3 5 0.8297

3 6 0.9795

3 7 1.0000

3 8 1.0000;

param gamma:=

1 1 0.00

1 2 0.13

1 3 0.36

1 4 0.83

1 5 0.94

1 6 0.96

1 7 0.96

1 8 0.98

2 1 0.17

2 2 0.93

2 3 1.0

2 4 1.0

2 5 1.0

2 6 1.0

2 7 1.0

2 8 1.0

3 1 0.0

3 2 0.7

3 3 0.9

3 4 1.0

3 5 1.0

3 6 1.0

3 7 1.0

3 8 1.0;

param M2:=

1 1 0.0

1 2 0.2

1 3 0.2

1 4 0.2

1 5 0.2

1 6 0.2

1 7 0.2

1 8 0.2

2 1 0.1317657

2 2 0.13667704

2 3 0.1317657

2 4 0.1317657

2 5 0.1317657

2 6 0.1317657

2 7 0.1317657

2 8 0.1317657

3 1 0.17020281

3 2 0.1727799

3 3 0.177838969

3 4 0.187838969

3 5 0.187838969

3 6 0.187838969

3 7 0.187838969

3 8 0.187838969;

param delta:=

1 1 0.000873979

1 2 0.000707626

1 3 0.00067365

1 4 0.00067365

1 5 0.00067365

1 6 0.00067365

1 7 0.00067365

1 8 0.00067365

2 1 0.000332387673

2 2 0.00023121717

2 3 0.000044811811

2 4 0.000044811811

2 5 0.000044811811

2 6 0.000044811811

2 7 0.000044811811

2 8 0.000044811811;

# individuals in 2010

param xstart:=

1 1 243.436

1 2 195.479

1 3 157.077

1 4 112.665

1 5 54.646

1 6 17.253

1 7 8.014

1 8 3.241

2 1 44.430

2 2 64.314

2 3 13.930

2 4 12.077

2 5 3.061

2 6 1.171

2 7 3.581

2 8 1.811

3 1 9806

3 2 7768

3 3 6928

3 4 2920

3 5 1815

3 6 745

3 7 901

3 8 1139;

**Profits of Baltic sea fisheries**

To determine the past profits for the Baltic fisheries on cod, herring, and sprat, we use data from STECF (<https://fishreg.jrc.ec.europa.eu/web/datadissemination>, accessed October 19, 2012) and from the ICES (2012) stock assessment report. STEFC provides cost data for countries and fleet segments. We use data for the Swedish and Polish fleets for the period 2008-2010. The reasons are that these fleets catch cod almost exclusively in the Baltic. Together they have about half the total quota for Eastern Baltic cod, the Swedish quota being 23%, and the Polish quota 26%, of the total allowable catch. We pool the data for the gear segments that comprise most of the catch: Demersal trawl and demersal seiner, drift nets and fixed nets, passive gears, pelagic trawl and seiner. The data we use is as follows:

| year | 2008 | 2009 | 2010 |
| --- | --- | --- | --- |
| variable cost [million EUR] | 119.14 | 97.32 | 117.55 |
| total landings value  [million EUR] | 149.42 | 122.78 | 123.28 |
| profit per value landed | 0.20 | 0.21 | 0.05 |
| cod price [EUR/kg] | 1.35 | 1.06 | 1.14 |
| cod landings [1000 tons] | 42.24 | 48.44 | 50.28 |
| cod profits [million EUR] | 11.53 | 10.60 | 2.66 |
| herring price [EUR/kg] | 0.34 | 0.32 | 0.33 |
| herring landings [1000 tons] | 126.16 | 134.13 | 136.71 |
| herring profits [million EUR] | 8.72 | 8.98 | 2.11 |
| sprat price [EUR/kg] | 0.15 | 0.14 | 0.16 |
| sprat landings [1000 tons] | 381.00 | 407.00 | 342.00 |
| sprat profits [million EUR] | 11.74 | 11.58 | 2.51 |

Variable costs are the sum of wages and salaries of crew, repair and maintenance costs, energy costs, other variable costs. Note that these are the variable cost for the entire fleet. To estimate profits for one species we adopt the approach of Kronbak (2005) and calculate the profits for that species by multiplying the profit margin ( (landings value – variable costs) divided by landings value) with the value of the respective species’ landings (price from STECF data multiplied by total landings in the Eastern/Central Baltic).

**References:**

EC (European Commission). 2007. Council Regulation (EC) No. 1098/2007 establishing a multi-annual plan for the cod stocks in the Baltic Sea and the fisheries exploiting those stocks, amending Regulation (ECC) No 2847/93 and repealing Regulation (EC) No 779/97.

Halpern BS et al. (2013) Achieving the triple bottom line in the face of inherent trade-offs among social equity, economic return and conservation. Proc Natl Acad Sci USA 110(15):6229:6234.

ICES (International Council for the Exploration of the Sea) (2012). Report of the Baltic Fisheries Assessment Working Group (WGBFAS). ICES CM 2012/ACOM:10

Quaas MF et al. (2012) Fishing Industry Borrows from Natural Capital at High Shadow Interest Rates. Ecological Economics 82:45–52

Quaas MF et al. (2013) Incentives for Optimal Management of Age-Structured Fish Populations. Res Ener Econ 35(2):113-134.

Ricker WE (1954) Stock and recruitment. Journal of the Fisheries Research Board of Canada 11:559-623

Spence AM (1974) Blue whales and applied control theory. In H. W. Gottinger (ed.), System Approaches and Environmental Problems (pp. 97–124) Göttingen: Vandenhoeck and Ruprecht.

Tahvonen O (2009) Economics of harvesting age-structured fish populations. Journal of Environmental Economics and Management 58 (3): 281-299

Tahvonen O, Quaas MF, Schmidt JO, Voss R (2013) Effects of species interaction on optimal harvesting of an age-structured schooling fishery. Environmental and Resource Economics 54(1):21-39.
