# Supplementary material for: Assessing Social – Ecological Trade-Offs to Advance Ecosystem-Based Fisheries Management
Source: PLoS One. 2014 Sep 30;9(9):e107811. doi: 10.1371/journal.pone.0107811 (PMC4182428; doi:10.1371/journal.pone.0107811)
Supplement: Materials S2 — Sensitivity analysis. Including Figure S1 (Relationship between the parameter of the welfare function [measured in Euros per kg of sprat spawning stock biomass] and the resulting steady-state spawning stock biomass of sprat), as well as Figure S2 (Relationship between the parameter of the welfare function and the resulting steady-state spawning stock biomass of sprat (a), and between the parameter of the welfare function and the resulting steady-state equity, as measured by 1 – the GINI coefficient. (DOCX) [file pone.0107811.s002.docx]

**Materials S2: Sensitivity analysis**

We systematically vary the two parameters$\lambda$ (the societal willingness to pay for non-fishery ecosystem services generated by the sprat stock) and $\theta$(the aversion against unequal incomes from the different fisheries) of the objective function. Figure S1 shows that an increasing $\lambda$results in an increased spawning stock biomass of sprat in the optimal steady state. Thus, there is a one-to-one relationship between $\lambda$ and sprat spawning stock biomass.

Figure S2 (a) shows the relationship between sprat spawning stock biomass in the optimal steady state and the parameter $\theta$ measuring the aversion against unequal fishing incomes from the three fisheries, and again we find a monotonic relationship. To report equity or inequity of fishing outcomes, we use the measure based on the Gini coefficient, as explained above. The results shown in Figure S2 (b) show that there is a positive one-to-one relationship between this equity measure evaluated in the optimal steady state and the parameter t we use in the objective function.

Error bars are determined (a) with respect to the major source of parameter uncertainty, standard errors of the parameters from the stock recruitment function and (b) with respect to uncertainty in predation mortalities. In both cases we performed a Monte-Carlo analysis as in Tahvonen et al. (2013).

(a) To assess the parameter uncertainty with respect to stock-recruitment functions, we generate 1,000 random parameter sets for the three pairs of parameters and , using the mean values and covariance matrix as derived from the statistical estimation of these parameters. When drawing random parameter values, we restrict the values of both and to be positive. For each parameter set, we compute the optimal steady state by numerically solving the first-order conditions at a steady state. The resulting values for spawning stock biomass, profits and fishing mortality are assumed to be log-normally distributed, and corresponding error bars (i.e. point result times the exponential function of $\pm$one standard deviation) are obtained from the standard deviation of the sample of results.

(b) To assess the parameter uncertainty with respect to predation mortality, the approach is similar. For each value of $\lambda$ and $\theta$ we generate a sample of 100 sets of predation mortalities drawing randomly and independently from a Normal distribution with mean equal to the point estimates of $\delta_{is}$ and variance equal to the squared standard errors obtained from the estimation. For each parameter set we determined steady-state values of the reported variables and use the standard deviations of the so obtained samples as standard errors.

*
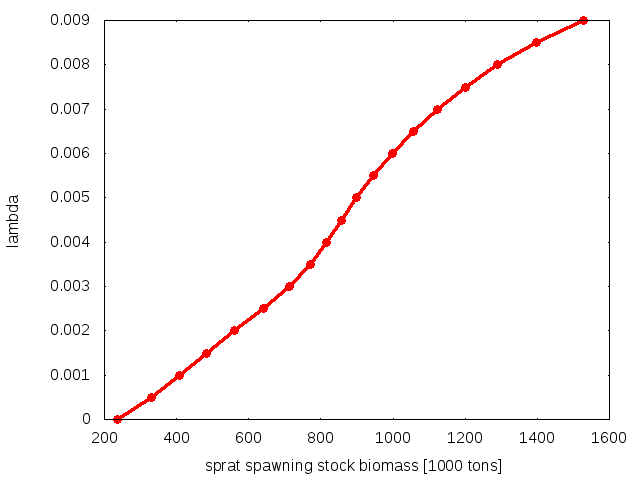
*

Figure S1. Relationship between the parameter $\lambda$ of the welfare function [measured in Euros per kg of sprat spawning stock biomass] and the resulting steady-state spawning stock biomass of sprat.


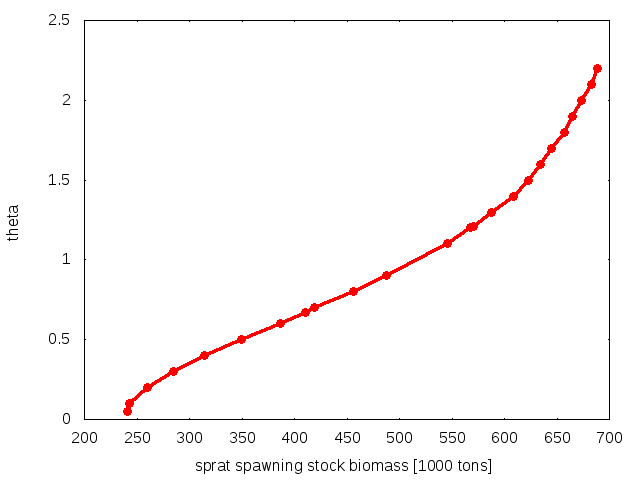

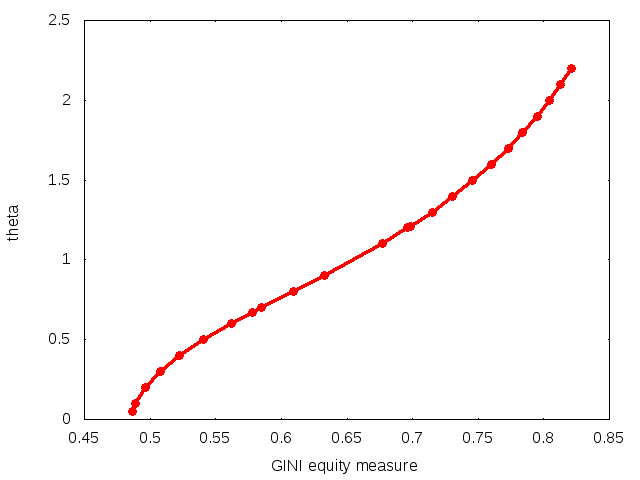


Figure S2. Relationship between the parameter $\theta$ of the welfare function and the resulting steady-state spawning stock biomass of sprat (a), and between the parameter $\theta$ of the welfare function and the resulting steady-state equity, as measured by 1 – the GINI coefficient .

**References:**

Tahvonen O, Quaas MF, Schmidt JO, Voss R (2013) Effects of species interaction on optimal harvesting of an age-structured schooling fishery. Environmental and Resource Economics 54(1):21-39.
