# Supplementary material for: Assessing Social – Ecological Trade-Offs to Advance Ecosystem-Based Fisheries Management
Source: PLoS One. 2014 Sep 30;9(9):e107811. doi: 10.1371/journal.pone.0107811 (PMC4182428; doi:10.1371/journal.pone.0107811)
Supplement: Materials S3 — Single versus multi-species management. Including Figure S3 (Timepath of optimal singlespecies management (red lines) in terms of spawning stock size (full line) and profit (dotted line) versus optimal multispecies management (blue lines); data from profit maximizing model run 2010-2040; trade-offs between cod and sprat (left panel), cod and herring (middle) and herring and sprat (right). (DOCX) [file pone.0107811.s003.docx]

**Material S3: Single versus multi-species management**

Considering trade-offs in multi-species fisheries in a transparent way requires analytical tools for assessing conflicts among fisheries such as our coupled ecological-economic model system. To demonstrate the advantages of the model system in predicting trade-offs to be made in fisheries management, we confronted the results to species by species optimization simulations using separate models for the three interacting species (Fig. S3). According to economic theory in fisheries, separate single-species optimization simulations result necessarily in high stock sizes and profits for all species. By accounting for species interactions, our multi-species model simulations clearly show that prioritizing the cod fishery would result in sacrificing ecological and economic potential in the sprat fishery, while focusing on the sprat fishery would result in losses in the cod and herring fisheries. Hence, our multi-species model system challenges traditional single-species approaches, and shows that optimal, long-term stock sizes and profits are significantly smaller compared to widely unrelated species by species simulations.


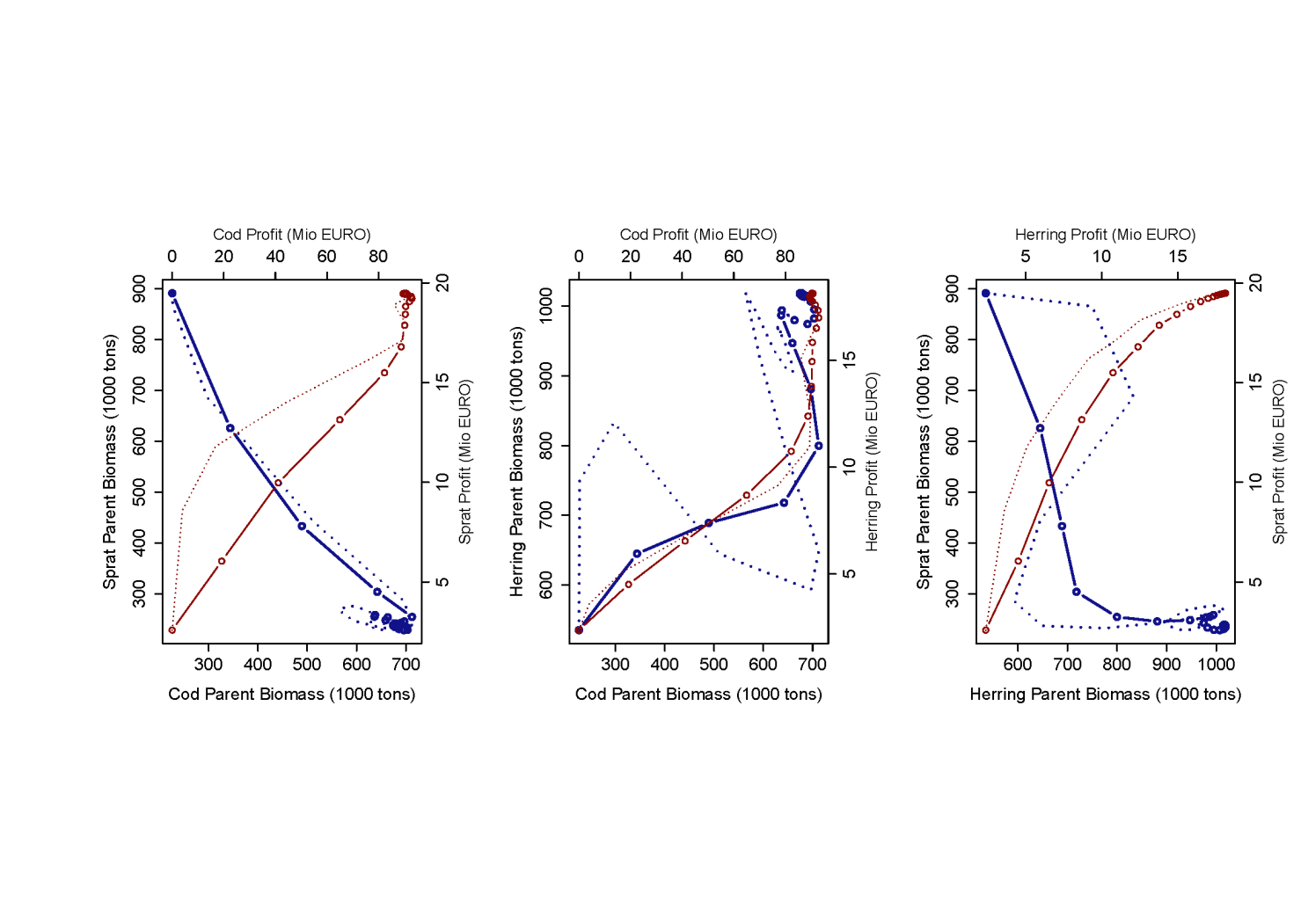


Figure S3. Singlespecies versus multispecies management. Timepath of optimal singlespecies management (red lines) in terms of spawning stock size (full line) and profit (dotted line) versus optimal multispecies management (blue lines); data from profit maximizing model run 2010-2040; trade-offs between cod and sprat (left panel), cod and herring (middle) and herring and sprat (right).
