# Supplementary material for: Assessing Social – Ecological Trade-Offs to Advance Ecosystem-Based Fisheries Management
Source: PLoS One. 2014 Sep 30;9(9):e107811. doi: 10.1371/journal.pone.0107811 (PMC4182428; doi:10.1371/journal.pone.0107811)
Supplement: Table S1 — Parameters of the multi-species ecological-economic model. Subscripts C, S, H refer to cod, sprat and herring, respectively. (DOCX) [file pone.0107811.s004.docx]

Table S1: Parameters of the multi-species ecological-economic model. Subscripts C, S, H refer to cod, sprat and herring, respectively.

|  |  | age group |  |  |  |  |  |  |  |  |  |
| --- | --- | --- | --- | --- | --- | --- | --- | --- | --- | --- | --- |
| parameter |  | 1 | 2 | 3 | 4 | 5 | 6 | 7 | 8 |  |  |
| maturities | $\gamma_{Cs}$ | 0 | 0.13 | 0.36 | 0.83 | 0.94 | 0.96 | 0.96 | 0.98 |  |  |
|  | $\gamma_{Cs}$ | 0.17 | 0.93 | 1.0 | 1.0 | 1.0 | 1.0 | 1.0 | 1.0 |  |  |
|  | $\gamma_{Cs}$ | 0.0 | 0.7 | 0.9 | 1.0 | 1.0 | 1.0 | 1.0 | 1.0 |  |  |
| Nat. mortality | *M_2Cs_* | 0.0 | 0.2 | 0.2 | 0.2 | 0.2 | 0.2 | 0.2 | 0.2 |  |  |
|  | *M_2Ss_* | 0.1318 | 0.1368 | 0.1318 | 0.1318 | 0.1318 | 0.1318 | 0.1318 | 0.1318 |  |  |
|  | *M_2Hs_* | 0.1702 | 0.1728 | 0.1778 | 0.1878 | 0.1878 | 0.1878 | 0.1878 | 0.1878 |  |  |
| Pred. mortality | $\delta_{Ss}$ | 0.873979 | 0.707626 | 0.67365 | 0.67365 | 0.67365 | 0.67365 | 0.67365 | 0.67365 |  |  |
| [1/mill. tons] | $\delta_{Hs}$ | 0.332388 | 0.231217 | 0.04481 | 0.04481 | 0.04481 | 0.04481 | 0.04481 | 0.04481 |  |  |
| Weights [kg/indiv.] | $w_{Cs}$ | 0.08 | 0.187 | 0.698 | 0.85 | 1.022 | 1.258 | 2.218 | 3.792 |  |  |
| [kg/1000 indiv.] | $w_{Ss}$ | 5.2 | 8.0 | 9.9 | 10.7 | 11.0 | 11.2 | 10.8 | 11.4 |  |  |
| [kg/indiv.] | $w_{Hs}$ | 0.012 | 0.0183 | 0.0258 | 0.0322 | 0.0332 | 0.0385 | 0.045 | 0.045 |  |  |
| catchability | $q_{Cs}$ | 0.0 | 0.1234 | 0.5651 | 1.0000 | 0.9776 | 0.7797 | 0.6389 | 0.6389 |  |  |
|  | $q_{Ss}$ | 0.4028 | 0.7695 | 0.8580 | 0.9643 | 0.8919 | 0.9965 | 1.0000 | 1.0000 |  |  |
|  | $q_{Hs}$ | 0.1929 | 0.3896 | 0.6111 | 0.7881 | 0.8297 | 0.9795 | 1.0000 | 1.0000 |  |  |
| Price | $p_{Cs}$ | 0.0 | 0.35 | 0.35 | 0.35 | 0.477 | 0.477 | 0.636 | 0.731 |  |  |
| Initial stock | $x_{Cs0}$ | 243.436 | 195.479 | 157.077 | 112.665 | 54.646 | 17.253 | 8.014 | 3.241 |  |  |
| Numbers | $x_{Ss0}$ | 44.430 | 64.314 | 13.930 | 12.077 | 3.061 | 1.171 | 3.581 | 1.811 |  |  |
|  | $x_{Hs0}$ | 9806 | 7768 | 6928 | 2920 | 1815 | 745 | 901 | 1139 |  |  |
