# Supplementary material for: Assessing Social – Ecological Trade-Offs to Advance Ecosystem-Based Fisheries Management
Source: PLoS One. 2014 Sep 30;9(9):e107811. doi: 10.1371/journal.pone.0107811 (PMC4182428; doi:10.1371/journal.pone.0107811)
Supplement: Table S2 — Biomass limits using the precautionairy approach (Bpa) and limit biomass levels (Blim) as well as different target fishing mortality rates depending on availability. (DOCX) [file pone.0107811.s005.docx]

**Table S2: Biomass limits using the precautionairy approach (Bpa) and limit biomass levels (Blim) according to ICES (2013) as well as different target fishing mortality rates depending on availability (Cod: EC 2007, sprat and herring: ICES 2012).**

|  | Management goal | | |
| --- | --- | --- | --- |
|  | B_pa_ | B_lim_ | F |
| Cod | 88 | 63 | 0.3 (F_ltmp_) |
| Sprat | 570 | 410 | 0.35 (F_MSY_) |
| Herring | 600 | 430 | 0.19 (F_pa_) |

**References**

EC (European Commission). 2007. Council Regulation (EC) No. 1098/2007 establishing a multi-annual plan for the cod stocks in the Baltic Sea and the fisheries exploiting those stocks, amending Regulation (ECC) No 2847/93 and repealing Regulation (EC) No 779/97.

ICES (International Council for the Exploration of the Sea) (2012). Report of the Baltic Fisheries Assessment Working Group (WGBFAS). ICES CM 2012/ACOM:10.

ICES (International Council for the Exploration of the Sea) 2013. Report of the Benchmark Workshop on Baltic Multispecies Assessments (WKBALT). ICES CM 2013/ACOM:43.
